# Supplementary material for: Persistent interferon signaling and clonal expansion mark early events in DNA methylation damage-induced liver cancer
Source: NAR Cancer. 2026 Jun 23;8(2):zcag014. doi: 10.1093/narcan/zcag014 (PMC13288108; doi:10.1093/narcan/zcag014)
Supplement: zcag014_Supplemental_Files [file zcag014_supplemental_files.zip › Supplemental Figures with Legends.pdf]

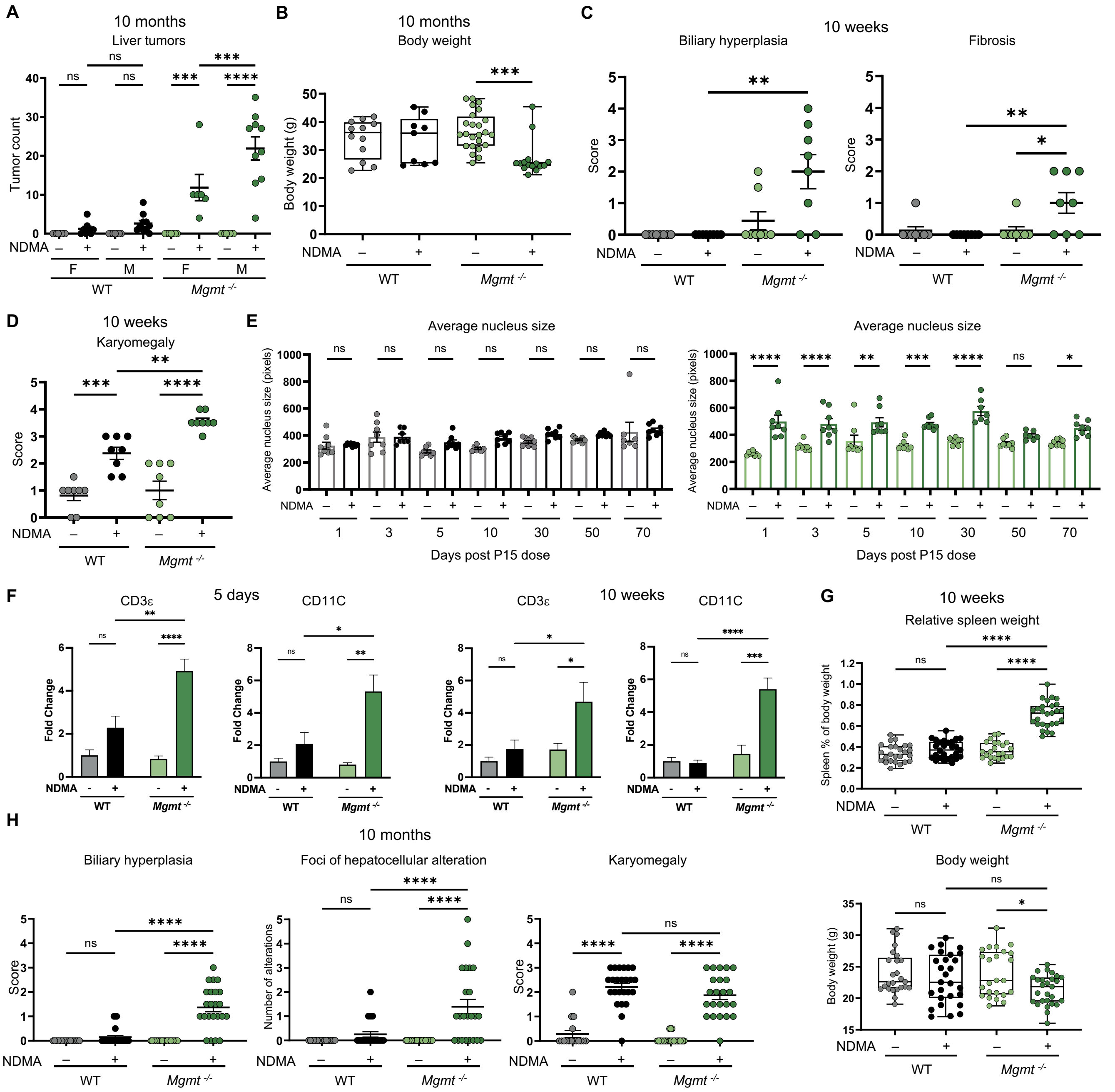

Figure S1. MGMT deficiency exacerbates NDMA-induced liver injury and tumorigenesis.
(**A**) Liver tumor counts at 10 months post-treatment in WT and *Mgmt*$^{-/-}$ mice, grouped by sex. $n \geq 6$ per group. (**B**) Mouse body weight at 10 months post-treatment (males and females combined). $n \geq 9$. Box plots indicate median, upper and lower quartiles, and whiskers showing maximum/minimum values. (**C**) Histopathological scores for liver morphologies, biliary hyperplasia and fibrosis, in WT and *Mgmt*$^{-/-}$ mice at 10 weeks post-treatment, assessed by H&E staining (sexes combined). $n \geq 8$. (**D**) Histopathological scores of karyomegaly in WT and *Mgmt*$^{-/-}$ mice at 10 weeks post-treatment, assessed by H&E staining (sexes combined). $n \geq 8$. (**E**) Quantification of mean nuclear size in liver cells by DAPI fluorescence. WT saline- (gray) and NDMA-treated (black). *Mgmt*$^{-/-}$ saline- (light green) and NDMA-treated (dark green). See Fig. 2C for images. $n \geq 7$. (**F**) Protein levels were measured via western blot to support T-cell activation (CD3ε & CD11C) in *Mgmt*$^{-/-}$ and WT livers at 5 days and 10 weeks post-exposure. $n = 4$ (2 males, 2 females). (**G**) Relative spleen weight (to body weight) and absolute body weight at 10 weeks post-treatment. $n \geq 23$. Box plots as described in (**B**). (**H**) Histopathological scores for biliary hyperplasia and karyomegaly and number of foci hepatocellular alterations in WT and *Mgmt*$^{-/-}$ mice at 10 months post-treatment, assessed by H&E staining (sexes combined). $n \geq 16$. Data are presented as mean ± s.e.m. Statistical comparisons performed using one-way ANOVA with Šídák's multiple comparisons test (**A**,**B**,**E**–**G**) and Kruskal-Wallis and Dunn's test (**C**,**D**,**H**). Statistical significance: *$p < 0.05$, **$p < 0.01$, ***$p < 0.001$, ****$p < 0.0001$. ns, not significant.

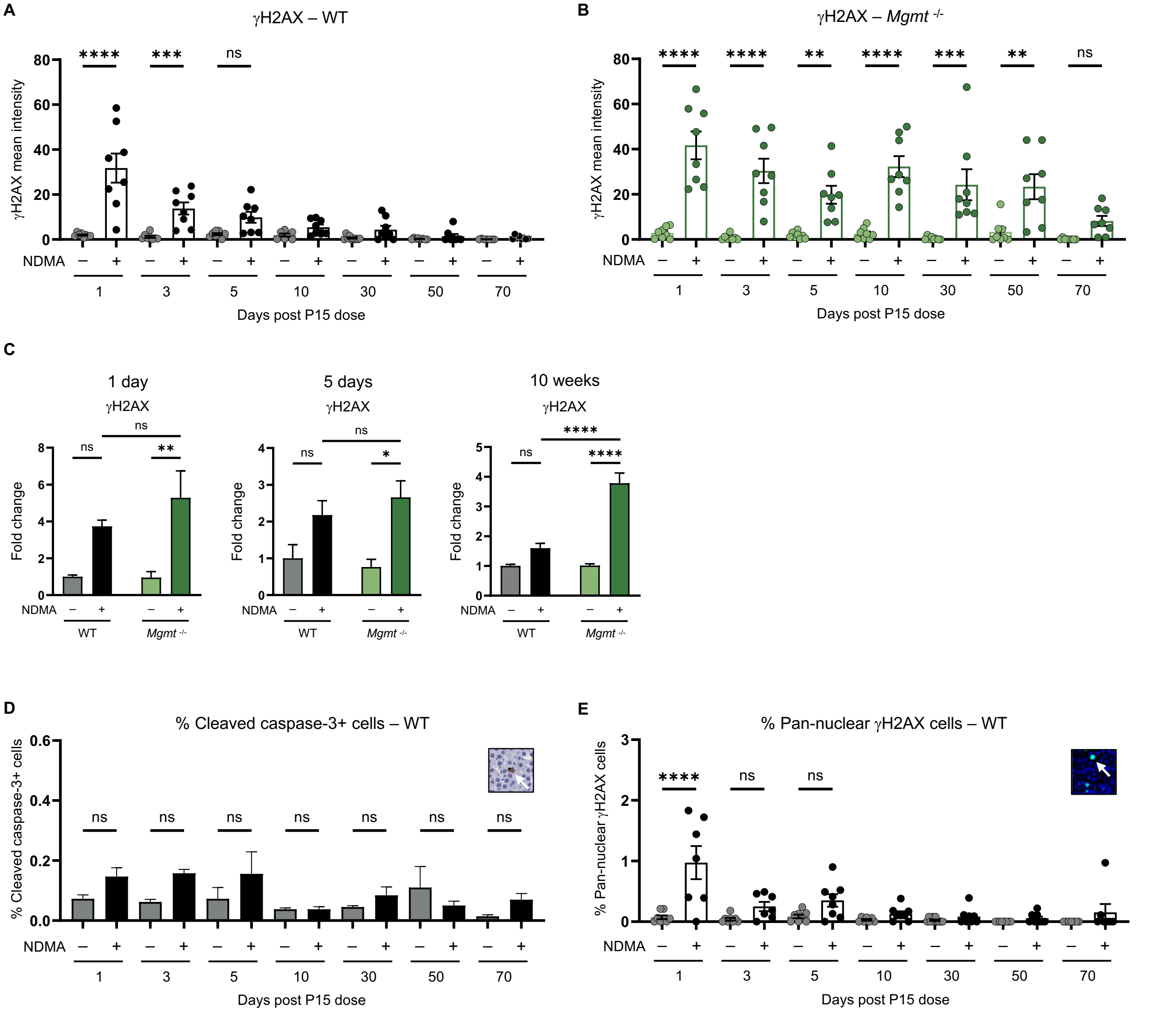

Figure S2. MGMT suppresses DNA damage retention, toxicity, and compensatory proliferation.

(**A**) Quantification of mean γH2AX fluorescence intensity per nucleus in WT livers, comparing saline-treated (gray) and NDMA-treated (black) groups. $n \geq 7$ per group.

(**B**) Quantification of mean gH2AX fluorescence intensity per nucleus in *Mgmt*$^{-/-}$ livers, comparing saline-treated (light green) and NDMA-treated (dark green) groups. $n \geq 7$ per group.

(**C**) Western blot analysis of gH2AX in whole-cell liver lysates at 1 day, 5 days, and 10 weeks post-exposure. Band intensities normalized to TPS and saline WT controls. $n = 4$ per group (2 males, 2 females).

(**D**) Quantification of apoptosis via cleaved caspase-3 positive cells as a percentage of total cells in WT livers at indicated timepoints, measured by IHC. Inset (white arrow) shows representative cleaved caspase-3 staining. $n \geq 7$.

(**E**) Quantification of apoptosis via immunofluorescence of pan-nuclear γH2AX-positive cells as a percentage of total cells in WT livers. Inset (white arrow) provides representative pan-nuclear γH2AX staining. $n \geq 7$.

Data are presented as mean ± s.e.m. Statistical comparisons performed using one-way ANOVA with Šídák's multiple comparisons test (**A**,**B**,**D**,**E**) and two-way ANOVA (**C**). Statistical significance: *$p < 0.05$, **$p < 0.01$, ***$p < 0.001$, ****$p < 0.0001$. ns, not significant.

A

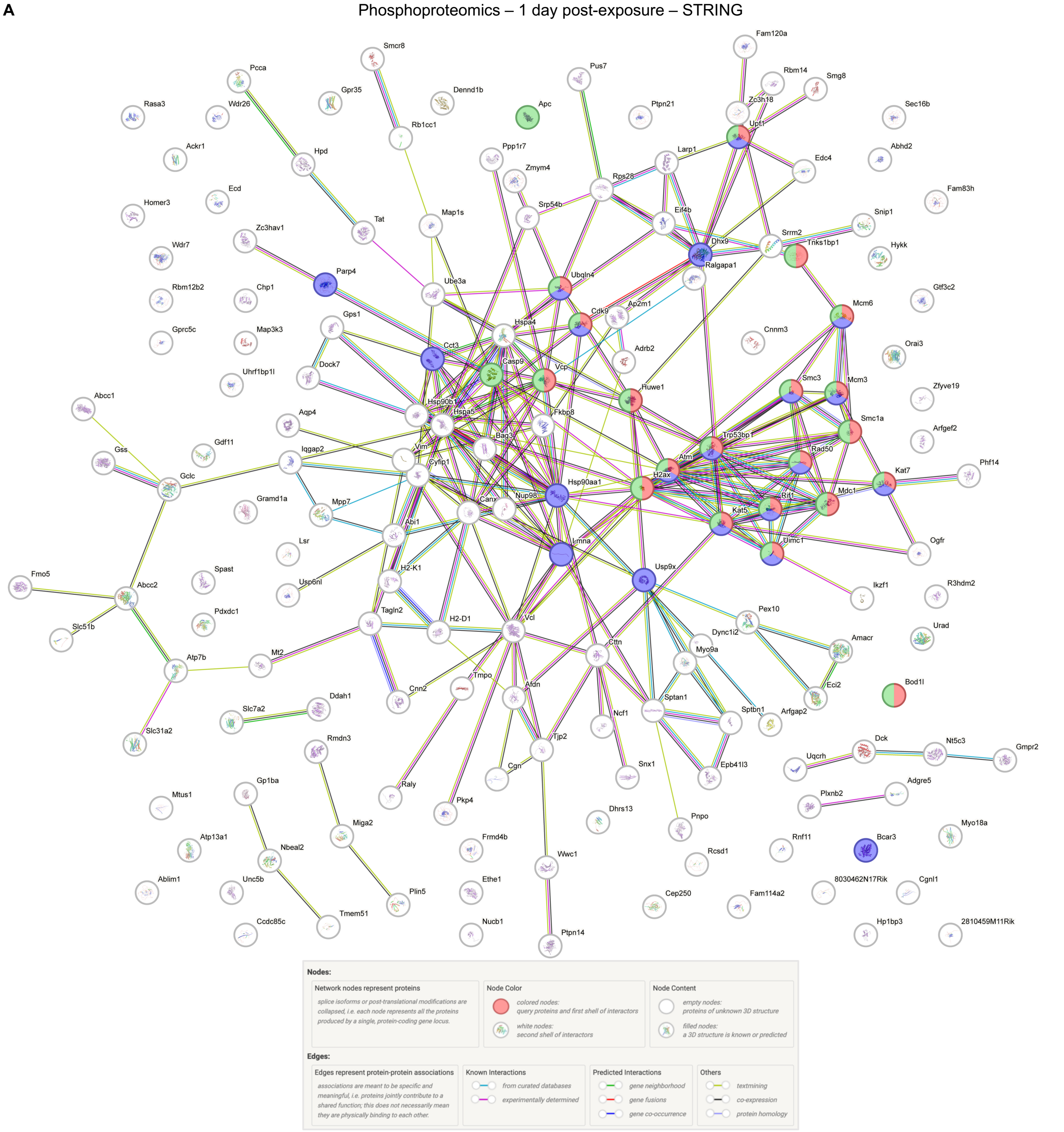

Figure S3. Immunoblotting and phosphoproteomics reveal an NDMA-induced DDR.
(**A**) STRING database analysis of top-expressed phosphoproteins at 1 day post-exposure. DNA repair clusters as shown in **Figure 3I** from biological processes in gene ontology are shown here. Colored nodes match the functional enrichments shown in the bar graph in **Figure 3I**: red = DNA repair, purple = regulation of DNA metabolic process, and green = cellular response to DNA damage stimulus. STRING key displayed for all other descriptions.

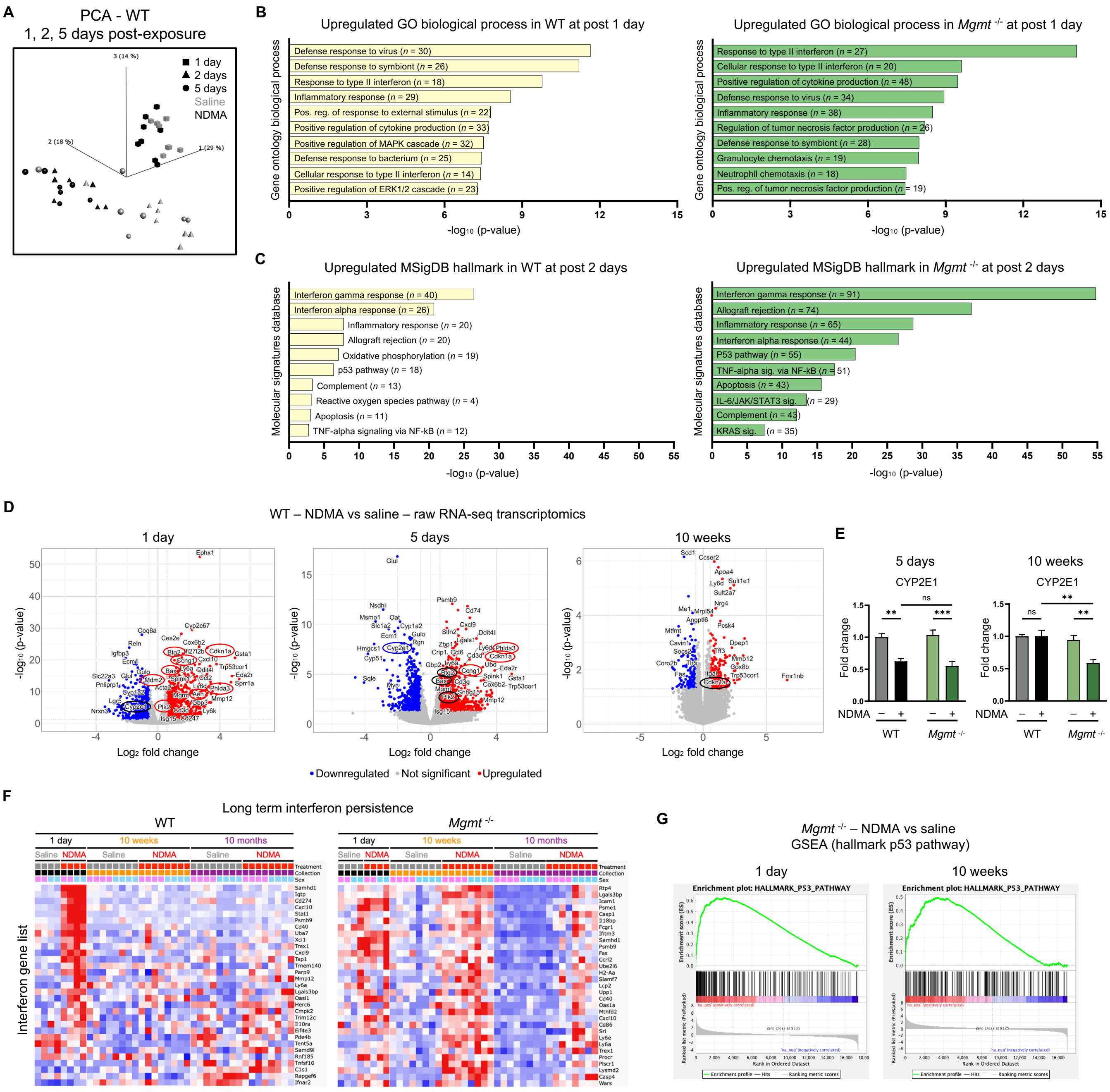

Figure S4. NDMA induces persistent transcriptome dysregulation and an IFN response. (**A**) PCA of WT liver samples collected at days 1, 2, and 5 post-saline or NDMA exposure. RNA was extracted from liver tissue and analyzed by RNA-seq; $n \geq 7$ per group (males and females combined). (**B**) Upregulated genes from NDMA-treated WT and *Mgmt*$^{-/-}$ liver samples (1 day post-exposure) were analyzed using Enrichr for Gene Ontology (GO) Biological Process enrichment. The top 10 enriched pathways are shown, ranked by Fisher exact test p-values. n = indicates number of genes in pathway. (**C**) Upregulated genes from NDMA-treated WT and *Mgmt*$^{-/-}$ liver samples (2 days post-exposure) were analyzed using Enrichr for MSigDB Hallmark gene set enrichment. The top 10 pathways are displayed, ranked by Fisher exact test p-values. (**D**) Volcano plots present differentially expressed genes in WT liver (NDMA vs saline) across selected timepoints (1 day, 5 days, and 10 weeks), with -log10 p-value plotted against $\log_2$ fold change. Genes circled in red belong to the GSEA Hallmark p53 pathway data set; Cyp2e1 is circled in blue. Differential expression was determined using an adjusted p-value cutoff of < 0.05 and absolute $\log_2$ fold change cutoff of > 0.58. (**E**) CYP2E1 protein abundance was assessed by western blot in liver samples at 5 days and 10 weeks post-exposure. Band intensities normalized to TPS and saline WT controls. n = 4 per group (2 males, 2 females). Statistical comparisons (two-way ANOVA with Šídák's multiple comparisons. Data are presented as mean ± s.e.m. Statistical significance: *p < 0.05, **p < 0.01, ***p < 0.001, ****p < 0.0001. ns, not significant. (**F**) Heatmaps display expression of a curated interferon response gene list in WT and *Mgmt*$^{-/-}$ livers at 1 day, 10 weeks, and 10 months post-exposure, demonstrating persistence of IFN signaling in *Mgmt*$^{-/-}$ samples. Sex: pink = female and light blue = male. (**G**) Gene Set Enrichment Analysis (GSEA) on *Mgmt*$^{-/-}$ liver at 1 day and 10 weeks post-NDMA exposure highlights enrichment of the Hallmark p53 pathway compared to saline controls.

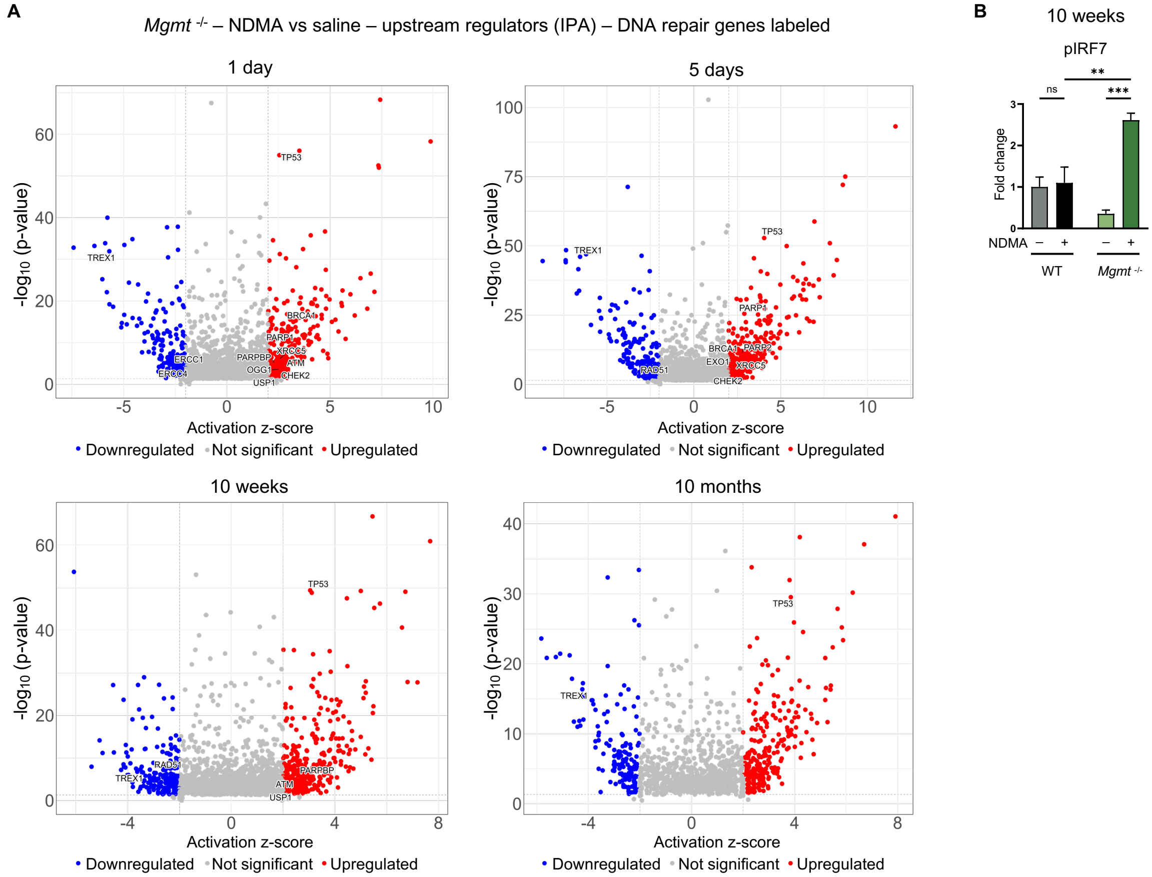

Figure S5. Upstream regulators of the IFN response are persistently activated in *Mgmt*$^{-/-}$ mice.

(**A**) Ingenuity Pathway Analysis (IPA) of RNA-sequencing data from *Mgmt*$^{-/-}$ mice identified few activated (> 2.0 z-score) and inhibited (< 2.0 z-score) upstream regulators throughout their lifetime pertaining to DNA repair genes (labeled in each plot). Analysis included transcripts with p-adjusted values < 0.05 and $\log_2$ fold change > 0.58.

(**B**) Phosphorylated IRF7 (pIRF7) protein levels were measured via western blot at 10 weeks post-exposure. Band intensities normalized to TPS and saline WT controls. $n = 4$ per group (2 males, 2 females). Statistical comparisons performed by two-way ANOVA with Šídák's multiple comparisons test. Data are presented as mean ± s.e.m. Statistical significance: *p < 0.05, **p < 0.01, ***p < 0.001, ****p < 0.0001. ns, not significant.

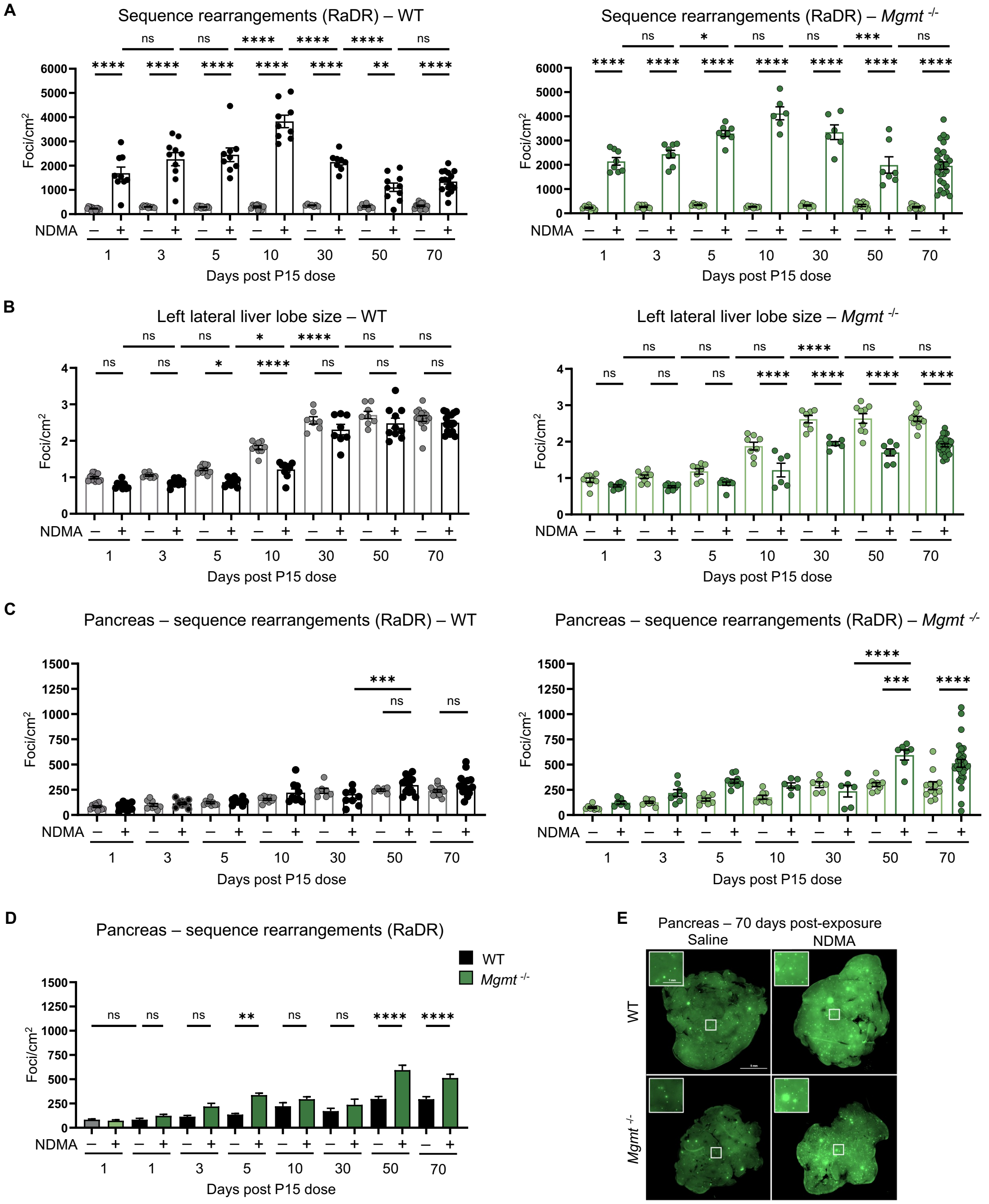

Figure S6. NDMA induces recombination events indicative of persistent genomic instability. (**A**) Quantification of sequence rearrangement mutations in liver samples of WT (left, gray/black bars) and *Mgmt*$^{-/-}$ (right, green bars) mice following NDMA or saline exposure at multiple timepoints, shows a peak at 10 days. RaDR foci (eGFP expression) per cm² quantified using machine learning image analysis. $n \geq 10$ per group. (**B**) Measurements of total left lateral liver lobe size (cm²) in WT (left) and *Mgmt*$^{-/-}$ (right) mice across NDMA and saline groups. Significant reductions are observed in NDMA-treated WT mice at 5 and 10 days, and in *Mgmt*$^{-/-}$ mice from 10 to 70 days post-exposure. $n \geq 10$ per group. (**C**) Quantitation of sequence rearrangement mutations in the pancreas, shown for WT (left) and *Mgmt*$^{-/-}$ (right) mice. RaDR foci per cm² quantified by machine learning. Significant increases are detected in *Mgmt*$^{-/-}$ mice after NDMA exposure at 50- and 70-days post-dose, but not in WT controls. $n \geq 10$ per group. (**D**) Direct comparison of pancreatic sequence rearrangement mutations post-NDMA exposure reveals significantly increased RaDR foci per cm² in *Mgmt*$^{-/-}$ (green bars) compared to WT (gray/black bars) at 50 and 70 days. $n \geq 10$ per group. (**E**) Representative whole-mount fluorescence images of pancreatic tissue from WT and *Mgmt*$^{-/-}$ RaDR-GFP mice 70 days after NDMA or saline treatment. Insets show higher magnification (scale = 1 mm) of boxed regions (scale = 5 mm at 2x) where areas are with GFP-positive foci indicating mutations. Statistical comparisons performed using one-way ANOVA with Šídák's multiple comparisons test (**A**–**D**). Data are presented as mean ± s.e.m. Statistical significance: *$p < 0.05$, **$p < 0.01$, ***$p < 0.001$, ****$p < 0.0001$. ns, not significant.

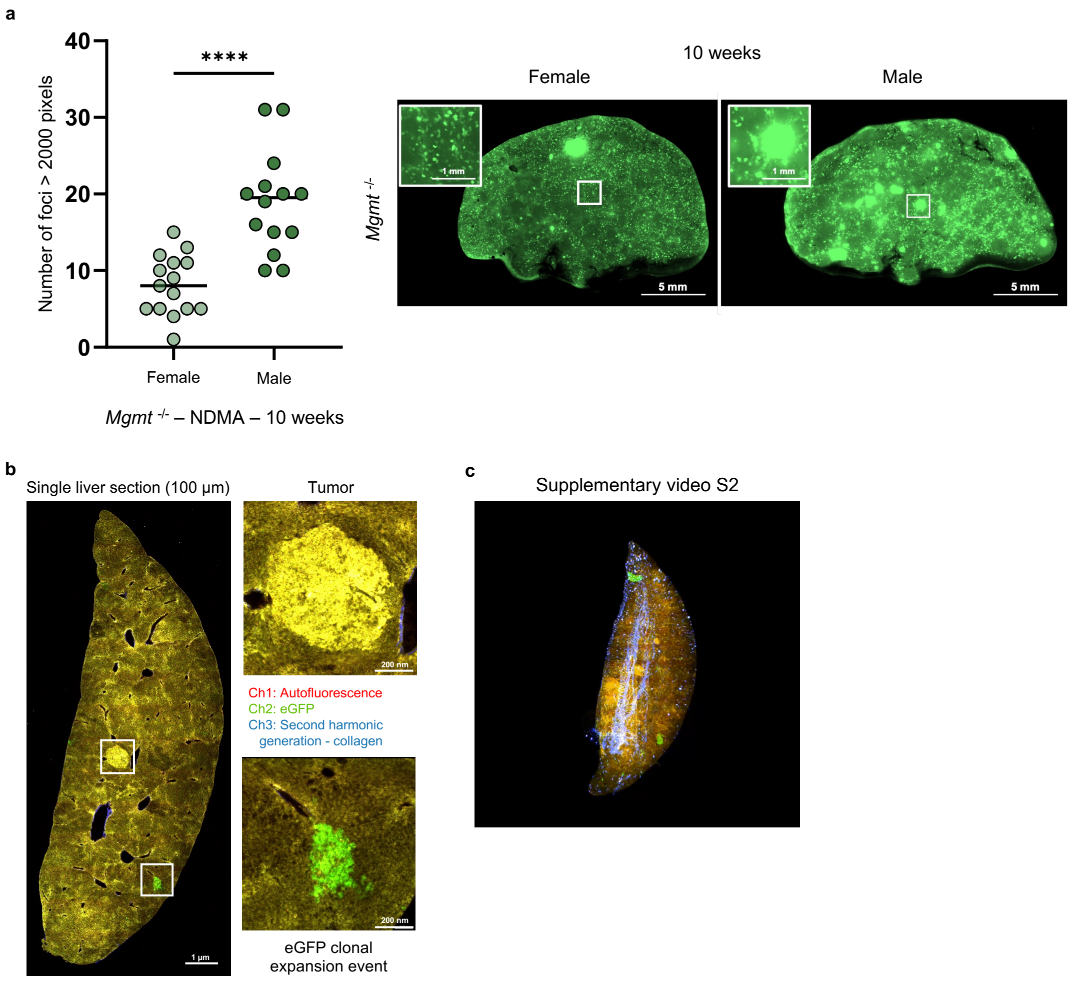

Figure S7. NDMA induces clonal expansion in *Mgmt*$^{-/-}$ livers.

(**A**) At 10 weeks post-NDMA exposure large clonal expansion events are significantly greater in male *Mgmt*$^{-/-}$ livers compared to females. A single large clonal expansion event was defined as RaDR-GFP foci exceeding 2000 pixels in area. Representative whole-mount eGFP images highlight the significant difference between female and male *Mgmt*$^{-/-}$ livers. $n \geq 14$ per group. Statistical comparisons performed using Mann-Whitney test. Data are presented as mean ± s.e.m. Statistical significance: *$p < 0.05$, **$p < 0.01$, ***$p < 0.001$, ****$p < 0.0001$. ns, not significant.

(**B**) 2-photon microscopy of a liver section (100 µm thick) from a ~13-month post-NDMA *Mgmt*$^{-/-}$ female mouse visualizes both eGFP-positive clonal expansion events (green, lower boxes) and tumor areas detected by tissue autofluorescence (yellow, upper box). Insets display high-magnification images of the indicated regions.

(**C**) 3-dimensional reconstruction of sequential 2-photon images from (**B**) visualizes the spatial distribution of eGFP clonal expansion events (green) and tumors (yellow) within a *Mgmt*$^{-/-}$ liver. The vascular network is displayed in blue. See **Supplementary Video S2**.

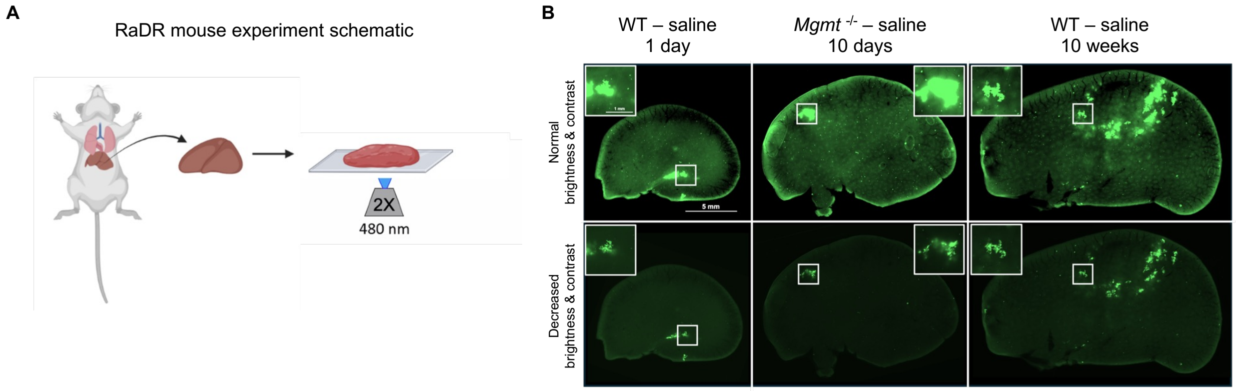

Figure S8. RaDR-GFP imaging.

(**A**) Schematic illustration of the RaDR-GFP mouse experiment. Freshly collected liver tissue is collected and mounted on a glass slide for GFP imaging at 2x magnification, with excitation at 480 nm. This protocol enables direct visualization of recombination reporter signals in liver tissue.

(**B**) Representative GFP fluorescence images of whole livers from WT and *Mgmt*$^{-/-}$ RaDR mice at 1 day, 10 days, and 10 weeks post-saline treatment, imaged at consistent exposure and emission settings. In rare cases (~2%), spontaneous recombination of the direct repeat substrate occurs early in development which can lead to EGFP expression in many daughter cells. Early recombination events can be distinguished from the majority by the presence of a jagged edge with a decrease in brightness and contrast. Out of 327 mice, this was observed in 7 samples, and these were eliminated from analysis. Insets highlight regions of interest.

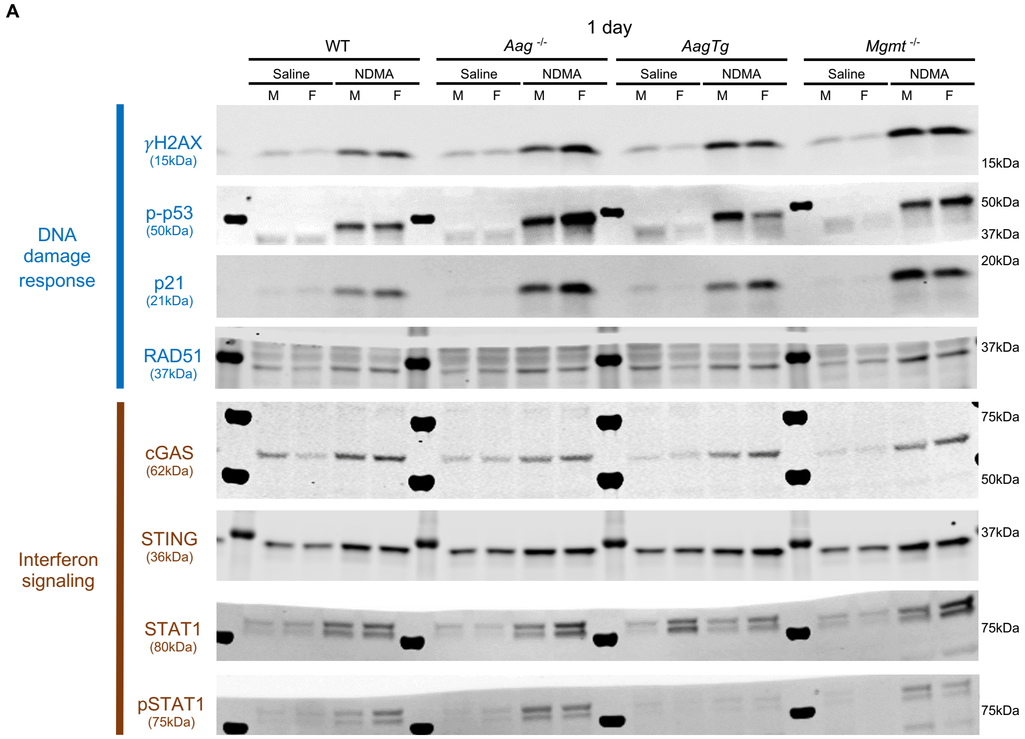

Figure S9. Western Blot Images.
(**A**) Whole-cell lysates from liver tissue collected at 1 day post-exposure from WT and *Mgmt*$^{-/-}$ mice (analyzed in this study) and from *Aag*$^{-/-}$ and *AagTg* mice (included for a separate project) were analyzed by western blot. Only WT and *Mgmt*$^{-/-}$ samples were discussed in the present manuscript. DNA damage response proteins for γH2AX (**Supplementary Figure S2C**), p-p53, p21, and RAD51 (**Figures 3A–3C**). Interferon signaling proteins for cGAS, STING, STAT1, and p-STAT1 (**Figure 5D**). Densitometry images provided. M = Male and F = Female.

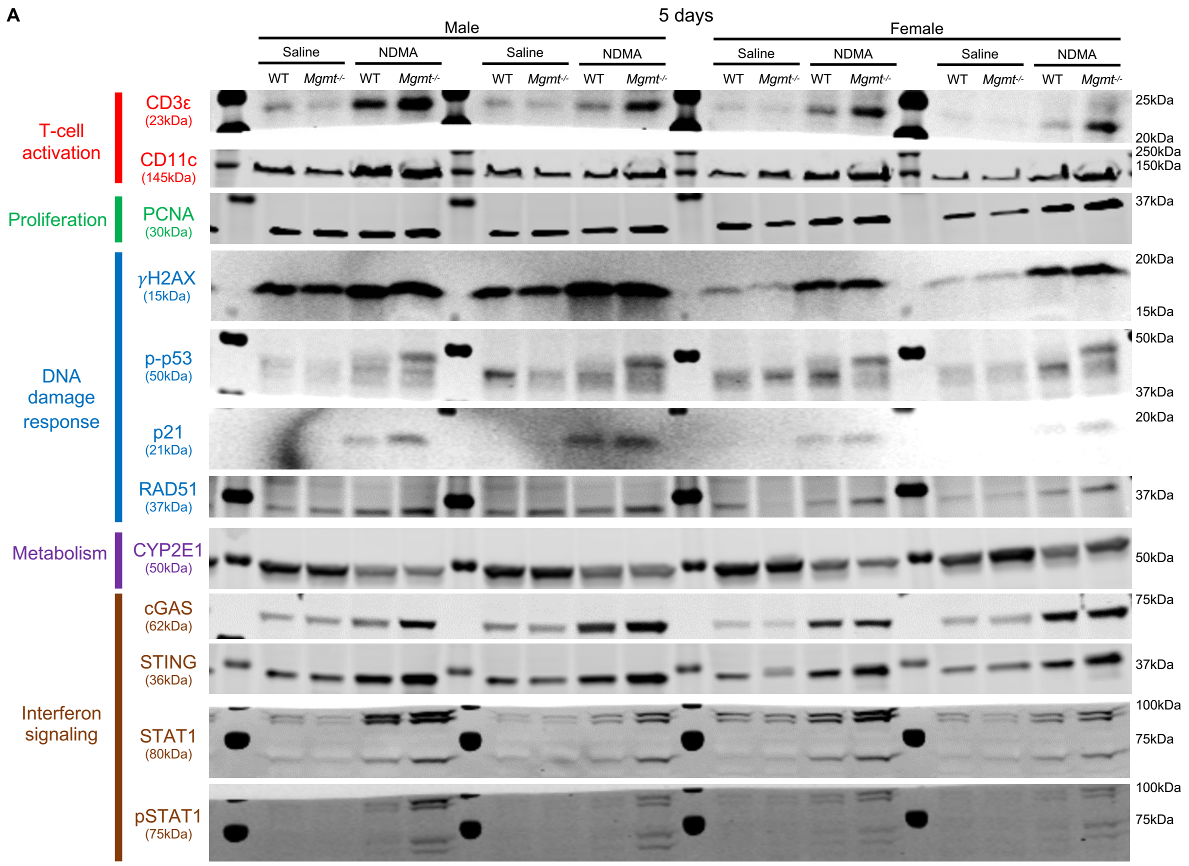

Figure S10. Western Blot Images.
(**A**) Whole-cell lysates from liver tissue collected at 5 days post-exposure from WT and *Mgmt*$^{-/-}$ mice were analyzed by western blot. T-cell activation proteins for CD3ε and CD11c (**Supplementary Figure S1D)**. Proliferation protein for PCNA (**Figure 2H**). DNA damage response proteins for γH2AX (**Supplementary Figure S2C**), p-p53, p21, and RAD51 (**Figures 3A–3C**). Metabolism protein for CYP2E1 (**Supplementary Figure S4D**). Interferon signaling proteins for cGAS, STING, STAT1, and p-STAT1 (**Figure 5D**). Densitometry images provided.

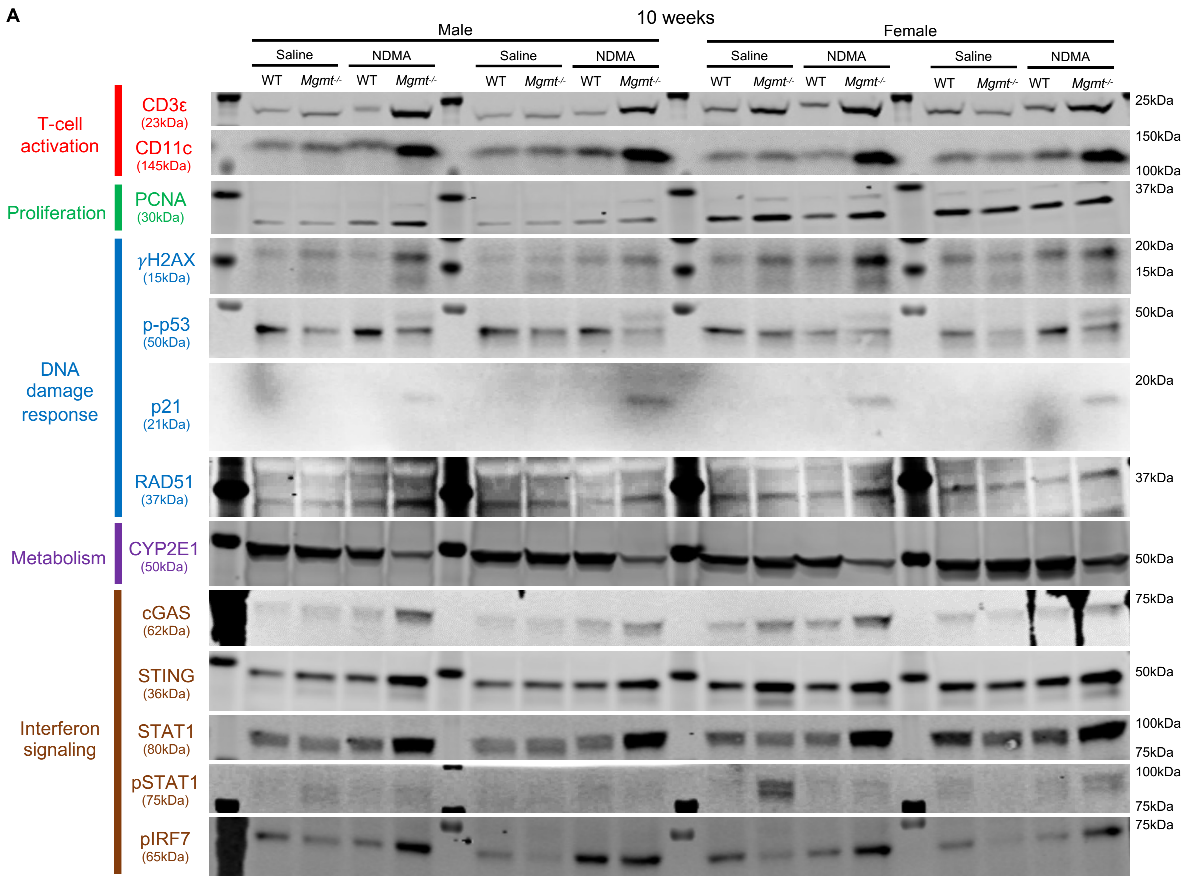

Figure S11. Western Blot Images.
(**A**) Whole-cell lysates from liver tissue collected at 10 weeks post-exposure from WT and *Mgmt*$^{-/-}$ mice were analyzed by western blot. T-cell activation proteins for CD3ε and CD11c (**Supplementary Figure S1D)**. Proliferation protein for PCNA (**Figure 2H**). DNA damage response proteins for γH2AX (**Supplementary Figure S2C**), p-p53, p21, and RAD51 (**Figures 3A–3C**). Metabolism protein for CYP2E1 (**Supplementary Figure S4D**). Interferon signaling proteins for cGAS, STING, STAT1, and p-STAT1 (**Figure 5D**), and pIRF7 (**Supplementary Figure S5B**). Densitometry images provided.
