# Supplementary material for: Persistent interferon signaling and clonal expansion mark early events in DNA methylation damage-induced liver cancer
Source: NAR Cancer. 2026 Jun 23;8(2):zcag014. doi: 10.1093/narcan/zcag014 (PMC13288108; doi:10.1093/narcan/zcag014)
Supplement: zcag014_Supplemental_Files [file zcag014_supplemental_files.zip › Supplementary Video 1-2 Legends.docx]

**Supplementary Video S1.** 3D reconstruction of a ~13-month post NDMA treated *Mgmt*^-/-^ female left liver lobe using sequential 2-photon images allows visualization of the spatial distribution of eGFP clonal expansion events (green), tumors (yellow) and the vascular network (blue).

**Supplementary Video S2.** 3D reconstruction of a ~13-month post NDMA treated *Mgmt*^-/-^ female left liver lobe using sequential 2-photon images allows visualization of the spatial distribution of eGFP clonal expansion events (green), tumors (yellow) and the vascular network (blue).
